# Mechanism study of isoflavones as an anti-retinoblastoma progression agent

**DOI:** 10.18632/oncotarget.19365

**Published:** 2017-07-18

**Authors:** Qifeng Wu, He Bai, Chu-Long Huang, Yongming Zhang, Xiayun Zeng, Huan Wan, Wen Zuo, Hai-Ying Wang, Yi-Xin Zeng, Yan-Dong Wang

**Affiliations:** ^1^ National Cancer Center /Cancer Hospital, Chinese Academy of Medical Sciences and Peking Union Medical College, Beijing, P. R. China; ^2^ Department of Drug and Cosmetics Registration (Department of TCMs and Ethno- Medicines Supervision), China Drug and Food Administration, Beijing, P. R. China; ^3^ State Key Laboratory of Ophthalmology, Zhongshan Ophthalmic Center, Sun Yat-sen University, Guangzhou, P. R. China; ^4^ Guangdong Wholewin Technology Ltd., Foshan, P. R. China; ^5^ Beijing Hospital, Beijing, P. R. China; ^6^ Department of Experimental Research, Sun Yat-sen University Cancer Center, Guangzhou, P. R. China; ^7^ State Key Laboratory of Oncology in Southern China, Guangzhou, P. R. China

**Keywords:** isoflavones, retinoblastoma, mTOR pathway, cyclin E1

## Abstract

Isoflavones, bioactive soy compounds, are known to exhibit anticancer activities. The present study investigated the anticancer activities of isoflavones on human retinoblastoma Y79 cells *in vitro* and *in vivo*. An MTT cell viability assay showed that the half maximal inhibitory concentration value of isoflavones against human retinoblastoma Y79 cells is 1.23 ± 0.42 μmol/l. Flow cytometry analysis indicated that isoflavones blocked G1/S progression. Western blot analysis demonstrated that the mammalian target of rapamycin (mTOR) pathway in Y79 cells was inhibited by isoflavones, with a concomitant decrease in cyclin E1, which accounted for the isoflavone-mediated G1 phase arrest. Isoflavones also inhibited human retinoblastoma growth *in vivo*; western blot analysis showed inhibition of mTOR and downregulation of cyclin E1 in an isoflavone-treated xenograft mouse model. Together, these results illustrate that isoflavones inhibit retinoblastoma tumour growth *in vitro* and *vivo* and that inactivation of the mTOR pathway and downregulation of cyclin E1 is involved in this action. The results of this study suggest that isoflavones could be tested as promising anti-retinoblastoma agent.

## INTRODUCTION

Retinoblastoma is the most commonly occurring intraocular malignant tumour in infants and young children and originates from photoreceptor precursor cells [1]. Chemotherapy has become the most common eye-sparing treatment modality [2]. However, present chemotherapy treatments result in noteworthy complications including second primary malignancies (e.g. acute myeloid leukaemia, bone tumours and leiomyosarcomas); this effect is best described for topoisomerase inhibitors and alkylating agents [3, 4]. Therefore, it is urgent to identify new therapeutic strategies to improve the clinical outcome of patients with retinoblastoma [5].

Genistein, daidzein, and glycitein are three major isoflavones which have some anti-cancer potential activities [6]. Genistein is cancer preventive phytochemical found in soy and other legumes [7-9] . Phase I clinical trials showed minimal toxicity in subjects treated with purified soy unconjugated isoflavone mixture [10]. Several studies have demonstrated that *in vitro*, genistein could prohibit the growth of various cancer cell lines including breast cancer cells [11], lung cancer cells [12], and colorectal cancer cells [9]. In addition, genistein could boost bacteriophage-mediated cancer cell killing [13]. Isoflavones are also helpful in the treatment of certain cancers by inhibiting tyrosine kinase activity and regulating Akt, mitogen-activated protein kinase and other signalling pathways [13-17]. The mammalian target of rapamycin (mTOR) is a serine-threonine kinase that regulates cell growth, proliferation, survival, angiogenesis and autophagy [18-20]. The mTOR pathway is commonly deregulated in human malignancies [21], including retinoblastoma [22]. A study revealed that activation of mTOR could modulate some molecules via retinoblastoma protein phosphorylated, which also told us the mTOR signal pathway may be directly related to some retinoblastoma proteins [23]; Then another research focused on RY-2f, a chemically synthesized isoflavone analog, that could inhibit ovarian tumorigenesis. Scientists in this study demonstrated that the mechanism of anti-cancer role was through PI3K/Akt/mTOR signaling pathway [24]. So these clues reminded us that isoflavones may be efficient to retinoblastoma.

In this study, we investigated whether there were anticancer activities of isoflavones in human retinoblastoma Y79 cells or not, and then we primarily studied its effects on the mTOR pathway *in vitro* and *vivo*.

## RESULTS

### Effects of isoflavones on cell viability

**Figure 1:**
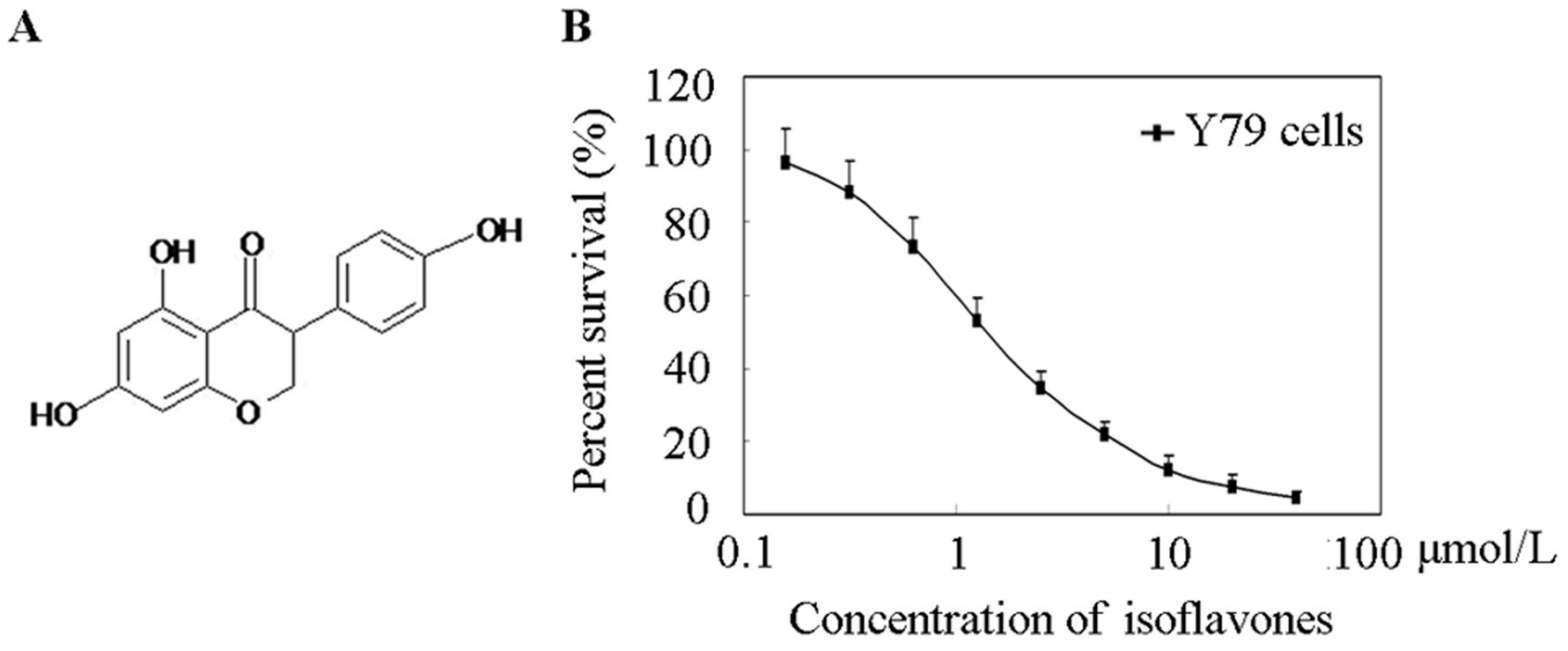
Effects of isoflavones on Y79 Cell proliferation **A.** Chemical structure of isoflavones. **B.** Cell viability was determined with MTT assay. Cells were exposed to the indicated concentrations of isoflavones for 48 h. Each point represents the mean ± standard error of three independent experiments.

The cytotoxic effect of isoflavones on human retinoblastoma Y79 cells was determined by MTT cell viability assay. Isoflavones inhibited the growth of the cells, as shown in Figure 1B. The IC_50_ value was 1.23 ± 0.42 μmol/L. The MTT assay showed that isoflavones were effective in blocking the proliferation of human retinoblastoma Y79 cells.

### Isoflavones induced G1 phase arrest

**Figure 2:**
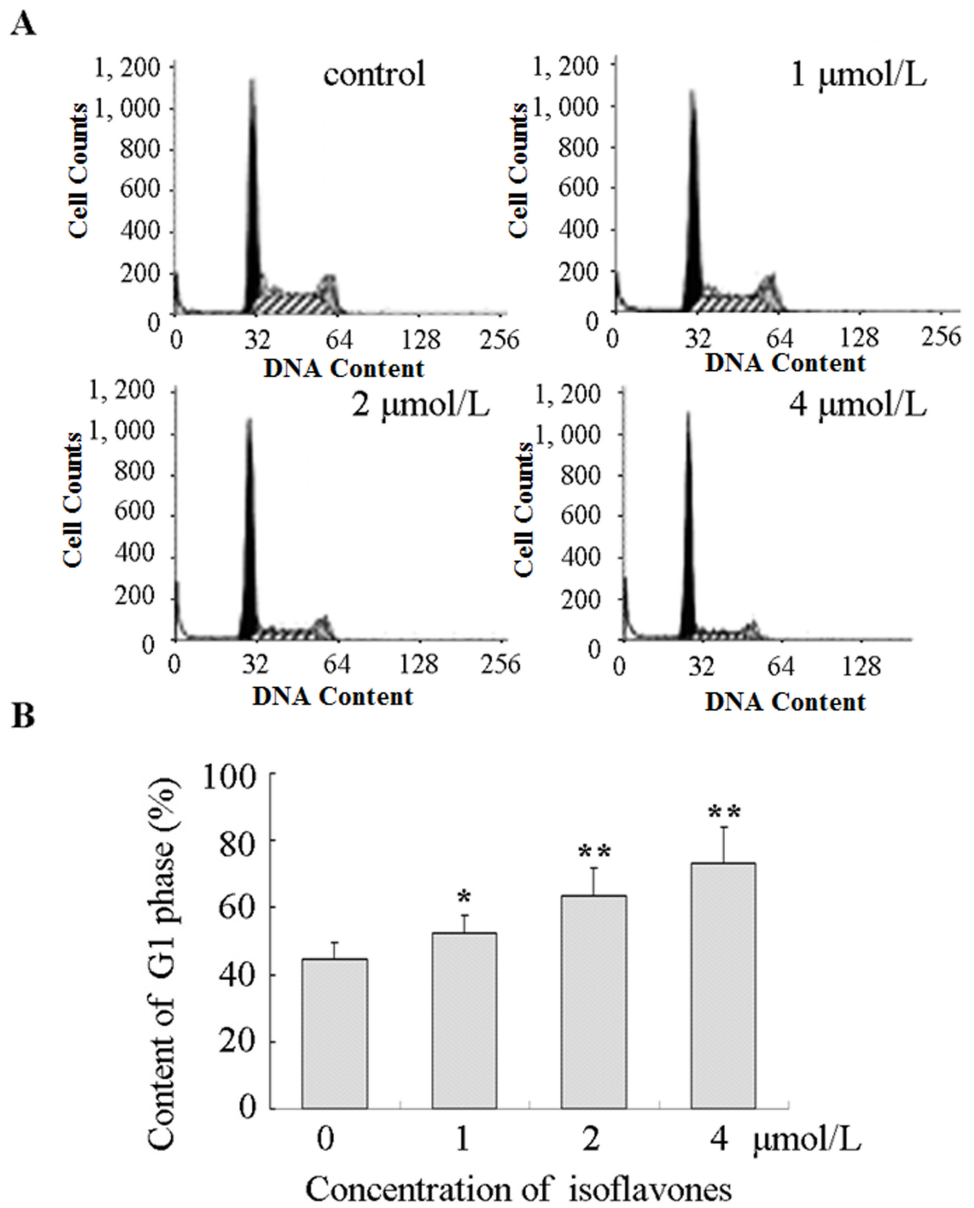
Isoflavones blocked G1/S progression in Y79 cells **A.** Cell cycle analysis was performed using propidium iodide staining and flow cytometry. All experiments were repeated at least three times, and a representative example of the DNA content is shown in frequency histograms. **B.** Cell cycle distribution. Columns: means of triplicate determinations; bars: standard deviations; *P < 0.05; **P < 0.01 compared with respective controls.

Flow cytometry was used to distinguish between cells in different phases of the cell cycle. G1 phase arrest was seen in cells treated with isoflavones, as shown in Figure 2A. The proportions of cells in the G1 phase after treatment with 0, 1, 2 and 4 μmol/l isoflavones were 44.53 ± 5.03%, 52.37 ± 5.24%, 63.73 ± 8.01% and 73.24 ± 10.98%, respectively (Figure 2B). Flow cytometry analysis showed that isoflavone treatment induced G1 arrest in a time-dependent manner which was associated with a concomitant significant.

### Isoflavones decreased phosphorylation of mTOR

**Figure 3:**
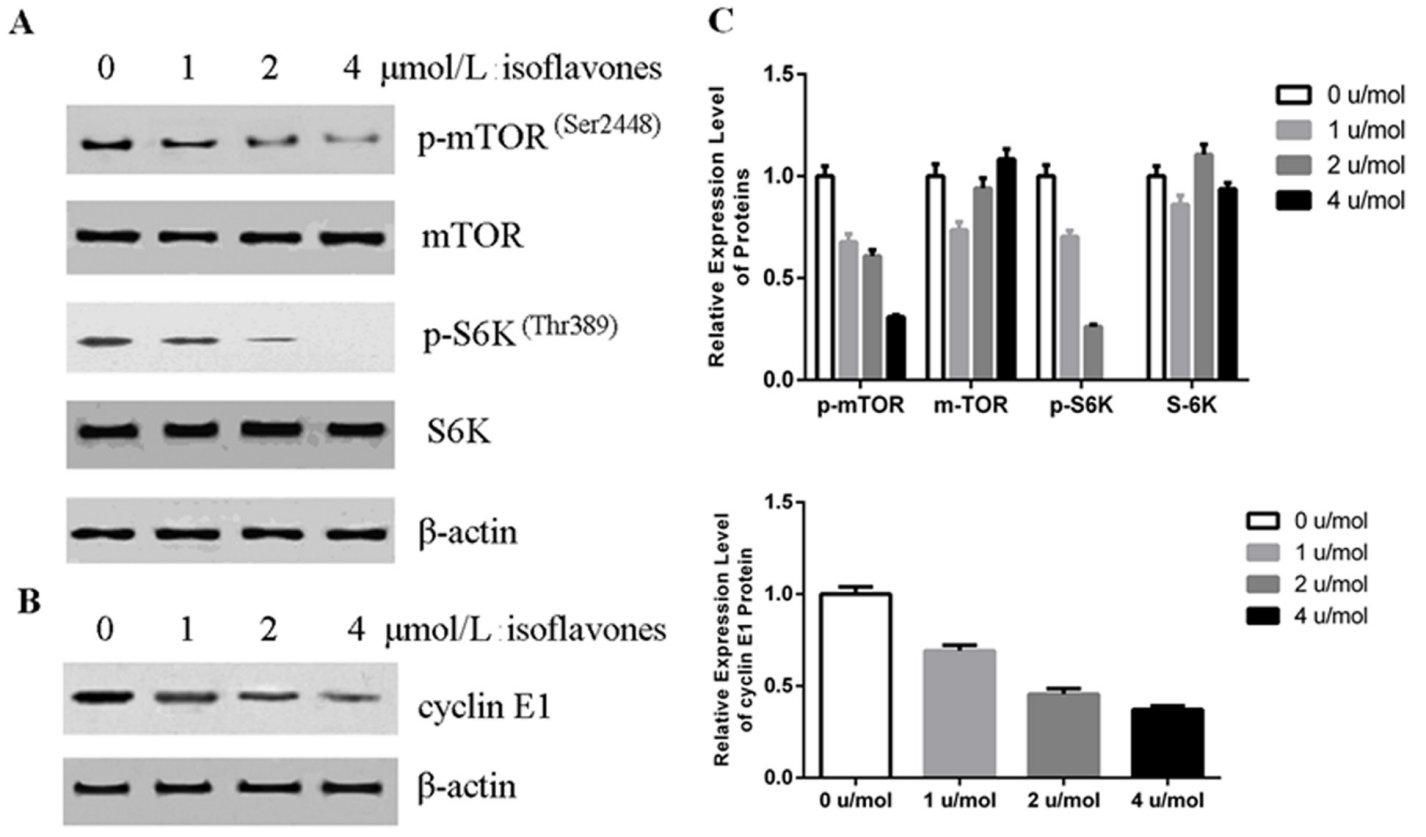
Isoflavones decreased phosphorylation of mTOR and cyclin E1 accumulation **A.** Isoflavones inhibited mTOR activity in Y79 cells as shown by western blot analysis. Y79 cells were treated with the indicated concentration of isoflavones for 48 h. **B.** Isoflavones decreased cyclin E1 protein in Y79 cells as shown by western blot analysis. Y79 cells were treated with the indicated concentration of isoflavones for 48 h. **C.** Bands of each protein were quantified by densitometric analysis and plotted after normalization against β-actin. Histogram shows means±SEM for three independent sets of experiments. *P* < 0.05.

Inhibition of the mTOR pathway can block the cell cycle between G0 and G1; this is indicated by a marked decrease in G1 cyclins in retinoblastoma cells [25]. mTOR activity is markedly increased in tumour cells and mTOR is highly phosphorylated in these cells [26]. We therefore investigated the phosphorylation of mTOR in human retinoblastoma Y79 cells after treatment with isoflavones with western blot analysis. As shown in Figure 3A, phosphorylated mTOR (p-mTOR) decreased in isoflavone-treated Y79 cells compared with that in control cells, without significant changes in mTOR protein. Ribosomal S6K is the best known downstream effector of mTOR and is also a direct substrate of mTOR [27]. p-S6K also decreased in cells treated with isoflavones without significant changes in S6K protein (Figure 3A). These results showed that isoflavones suppressed the mTOR pathway in Y79 cells.

### Isoflavones decreased cyclin E1 protein levels through the mTOR pathway

Cyclin E1 is a positive regulator that controls the transition of cells from G1 to S phase [28]. Numerous data have demonstrated that inhibiting mTOR decreases S6K phosphorylation, with a concomitant decrease in cyclin E1 levels [22, 29]. We therefore explored the effects of isoflavones on cyclin E1. As shown in Figure 3B, cyclin E1 decreased in cells treated with isoflavones. These results showed that isoflavones suppressed mTOR-mediated accumulation of cyclin E1.

### Isoflavones suppressed growth of human retinoblastoma xenografts *in vivo*

**Figure 4:**
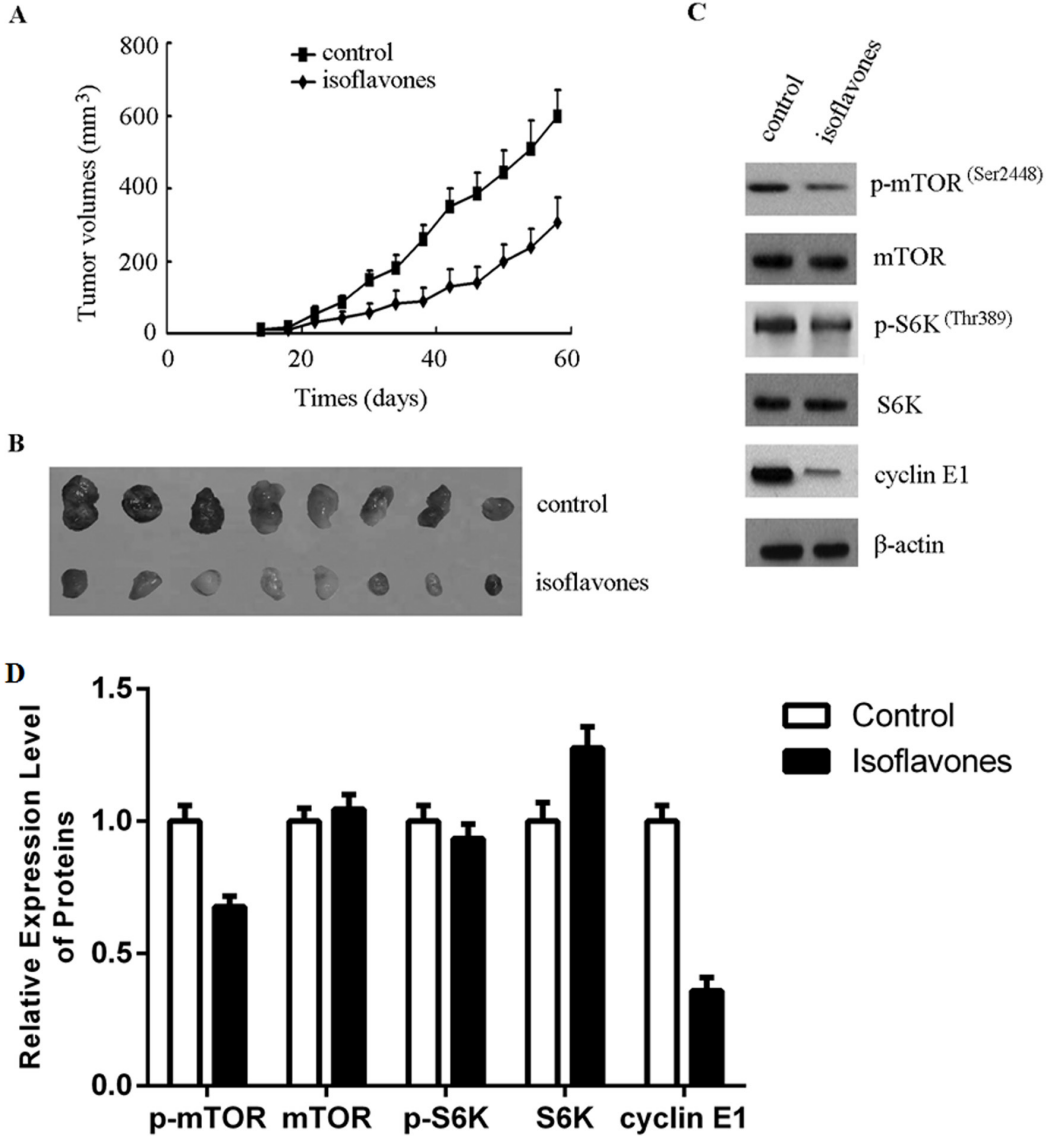
Isoflavones inhibited human retinoblastoma cell growth in a xenograft mouse model by decreasing the phosphorylation of mTOR and cyclin E1 accumulation **A.** Isoflavones inhibited human retinoblastoma cell growth in the xenograft mouse model. **B.** After euthanisation, the tumours were stripped and photographed. Tumours removed from isoflavone-treated mice are significantly smaller rather than control mice, showing the size difference between the tumours. **C.** Isoflavones decreased the phosphorylation of mTOR and cyclin E1 accumulation in xenograft tumour tissues. Total protein was extracted from tumour tissues. The indicated protein levels were determined with western blot analysis. **D.** Bands of each protein were quantified by densitometric analysis and plotted after normalization against β-actin. Histogram shows means ± SEM for three independent sets of experiments. *P* < 0.05.

In order to evaluate the *in vivo* effect of isoflavones on retinoblastoma growth, a xenograft mouse model of Y79 cells was established and these mice were treated with isoflavones or an equal volume of normal saline (control). As shown in Figure 4A, the mean tumour volume of the control group was larger than that of the isoflavone-treated group from day 18 (*P* < 0.05). Average tumor volume at 58 days was 308±66.5 mm^3^ in the isoflavone treatment group, whereas average tumor volume at 58 days was 603.5±79.8 mm^3^ in the control group. Similar results were also observed with tumor weight: The mean tumour weight of the isoflavone-treated group was 0.32 ± 0.06 g and that of the control group was 0.67 ± 0.09 g (*P* < 0.01, Figure 4B). These results showed that isoflavones significantly inhibited the growth of xenografted Y79 human retinoblastoma tumours in nude immune-deficient mice.

mTOR activity was assessed in the xenografted tumour tissue. As shown in Figure 4C and 4D, p-mTOR, p-S6K and cyclin-E1 expression was lower in the isoflavone-treated group than in the control group. These results showed that isoflavones also affected mTOR-mediated accumulation of cyclin E1 *in vivo*.

## DISCUSSION

Retinoblastoma is the most frequently occurring paediatric ocular cancer [30]. In developing countries, treatment is limited, and if untreated, the disease is always fatal. Long-term survival rates are low, and current chemotherapy causes significant morbidity in paediatric patients, which significantly limits dosing [31]. The mTOR pathway is one of the most commonly deregulated pathways in cancer and has been implicated in the tumorigenesis of retinoblastoma. Therefore, known inhibitors of this pathway are worth evaluating for the treatment of this disease [32]. Isoflavones, a group of phytoalexins isolated from soybean, have been known to exhibit anticancer activities, and investigations have suggested potent anticancer activities against multiple targets in the phosphatidylinositol-4,5-bisphosphate 3-kinase (PI3K)/Akt/mTOR pathway [33]. For example, isoflavones induced the suppression of S6K phosphorylation in oestrogen receptor-positive breast cancer cells [34] and decreased the phosphorylation of Akt and eIF4E proteins in human glioblastoma (U87) cells [35]. In this study, we found that isoflavones showed potent anticancer activity against human retinoblastoma Y79 cells *in vitro* and *in vivo* (Figures 1 and 4). Our investigation also elucidated that the isoflavone mechanism of action involved blockage of the mTOR pathway and decrease of cyclin E1.

The cell cycle is a vital process by which cells duplicated DNA and divide to produce daughter cells. Dysregulation of cell cycle components may lead to tumour formation [36]. In the present study, our results showed that isoflavones inhibited G1/S progression in Y79 cells (Figure 2). Cyclins are the key regulators of cell cycle progression. Cyclin E- cyclin-dependent kinase 2 has long been considered an essential master regulator of G1/S progression [37]. Cyclin E1 is a critical target of signals activated during tumorigenesis and is a well-established oncogene. The protein is unstable and is degraded by two distinct pathways involving the ubiquitin-proteasome system [38]; S58 in the N-terminal cluster is phosphorylated by glycogen synthase kinase 3 (GSK-3) [39].

Phosphorylation-triggered ubiquitination has been proposed to be the major pathway regulating the cyclin E1 protein, and mTOR is a critical regulator of GSK-3 activity [40]. mTOR has a key regulatory role in cell growth and homeostasis, and inhibition of mTOR is now a novel treatment strategy for several malignancies, either alone or in combination with other approaches [41]. A recent study also showed that cyclin E1 expression could be regulated by the PI3K/Akt/mTOR signalling pathway [29]. In the present study, inhibition of mTOR and downregulation of cyclin E1 occurred in isoflavone-treated human retinoblastoma Y79 cells and in an isoflavone-treated xenograft mouse model (Figures 3, 4C and 4D). This was supported by *in vitro* and *in vivo* data showing that isoflavones decreased mTOR phosphorylation, with a concomitant decrease in cyclin E1, which inhibited G1/S progression of human retinoblastoma Y79 cells.

The results *in vitro* and *in vivo* showed the isoflavones could inhibit human retinoblastoma Y79 cells. Also there were some other study results demonstrated good promises of isoflavones. Some researchers combined Flt1 peptide with isoflavones, which contributed to exploit this conjugated micelle-like nanoparticles to encapsulate isoflavones. The *in vivo* biological results showed statistically significant suppression of corneal neovascularization in cauterized corneas of Sprague-Dawley (SD) rats; the retinal vascular hyperpermeability significantly reduced in diabetic retinopathy model rats [42]. Then another research team use the diabetic retina rats to study isoflavone therapeutic effect. They treated the rats with isoflavones by subcutaneous injection. The results showed that isoflavones could ameliorate the histological changes of diabetic retinopathy in rats, which effects highly resembled a normal retina [43]. Thus, we can draw some preliminary inferences from the results above. Firstly, isoflavones possess good potential druggability. As previously mentioned, the novel combination of Flt1 peptide and isoflavones was feasible, which could be investigated for further clinical development. Secondly, isoflavones could treat some eye diseases such as diabetic retinopathy. This strongly demonstrated that isoflavones would play its therapeutic effect in eye not only for diabetic related eye disease. Lastly, isoflavones could enter in eyes via blood circulation, which provided another way for drug delivery. And this speciality offers us more possibility to develop its clinical use, especially for its use into eye drops in the future, which could reduce the side effects of general systemic administration. All these results above illustrated the great potential clinical application for isoflavones especially for eye diseases. These also provided us the molecular basis for further eye tumour studies.

In summary, the results of this study suggested that isoflavones reduced cell viability and triggered G1-phase blockage by down regulating cyclin E1 protein in human retinoblastoma Y79 cells *in vitro* and *in vivo*, mediated through the inhibition of the mTOR pathway. Although further studies are needed, these results have demonstrated the anticancer activities of isoflavones in human retinoblastoma cells and the mechanisms of action involved. Together, these results suggest that isoflavones could be a promising chemotherapeutic agent for retinoblastoma.

## MATERIALS AND METHODS

### Reagents and antibodies

Roswell Park Memorial Institute 1640 medium was purchased from Gibco BRL. Foetal bovine serum was obtained from Life Technologies Corporation. MTT, dimethyl sulfoxide, propidium iodide (PI) and other chemicals were purchased from Sigma Chemical Co. mTOR, phospho-mTOR (Ser2448), p70S6 kinase (S6K), phosphorylated S6K (p-S6K) (Thr389), cyclin D1 and β-actin antibodies were purchased from Cell Signaling Technology Inc. Genistein was bought from Sigma-Aldrich Co. LLC.

### Cell lines and cell culture conditions

The human retinoblastoma cell line Y79 was obtained from the American Tissue Type Culture Collection and cultured in Roswell Park Memorial Institute 1640 medium supplemented with 10% foetal bovine serum, 1% penicillin and 1% streptomycin. Y79 cells were grown at 37°C in a humidified incubator of 5% CO_2_ and 95% air, as previously described [44].

### Cell viability

Cell viability was determined with MTT assay. Briefly, Y79 cells were seeded in 96-well plates at a density of 3,000 cells/well and grown overnight. The next day, isoflavones diluted with growth medium at a full range of concentrations were added to the wells. After incubation for 48 h, 10 μl MTT (5 mg/ml) was added to each well and the plates were incubated at 37°C for an additional 4 h. The medium was then removed using a needle and syringe and dimethyl sulfoxide (100 μl) was added to each well and pipetted up and down to dissolve the insoluble purple formazan. Finally, the optical density was measured at wavelengths in the range of 500-600 nm using a plate reader (Thermo Fisher Scientific Inc.). The half maximal inhibitory concentration (IC_50_) values were calculated from survival curves as previously described [45]. All experiments were repeated at least three times.

### Cell cycle analysis

Cell cycle analysis was conducted using PI dyes and flow cytometry. Briefly, Y79 cells were seeded in 6-well plates at a density of 2 × 10^5^ cells/well and grown overnight. The next day, Y79 cells were treated with 0, 1, 2 and 4 μmol/L isoflavones for 48 h. After harvesting and washing, cells were fixed in ice-cold 70% ethanol at 4°C for 24 h. PI (400 µl, 50 µg/ml) was added for staining for 30 min. Finally, cell cycle phases (G0/G1, S, or G2/M) were analysed with flow cytometry using 488 nm excitation and 620 nm collection fluorescence. Cell cycle distribution was evaluated using MultiCycle AV software. All experiments were repeated at least three times.

### Western blot analysis

Western blotting was performed as previously described [44]. Briefly, Y79 cells were seeded in 6-well plates at a density of 2 × 10^5^ cells/well and grown overnight. The next day, cells were treated with isoflavones at the 0, 1, 2 and 4 μmol/L for 48 h. After harvesting and washing, cells were lysed for 5 minutes in cold radioimmunoprecipitation assay lysis buffer. Equivalent amounts of protein were separated on 8%-12% sodium dodecyl sulphate polyacrylamide gel electrophoresis gel and electrotransferred onto polyvinylidene difluoride (PVDF) membranes (Millipore, USA). PVDF membranes were blocked with 0.05% Tween 20 and 5% non-fat dry milk for 2 h to block nonspecific binding. They were then incubated with primary antibody and appropriate horseradish peroxidase-linked secondary antibodies. β-actin was used as an endogenous control. Finally, bands of specific proteins on the PVDF membranes were detected with western blotting Luminol reagent (Millipore, USA). All experiments were repeated at least three times.

### Xenograft mouse model

Specific pathogen-free BALB/c nude mice (weight, 18-20 g) were used as a xenograft mouse model. The research was conducted in accordance with the guide from the Review Committee for the Use of Human or Animal Subjects of Sun Yat-sen University and was approved by the Institution of Animal Care and Use Committee at the Institute of Biophysics, Chinese Academy of Sciences (SYXK2013-02). There were eight mice per group (treatment and control). Y79 cell suspension at a cell density of 1 × 10^7^ cells per 0.2 ml was subcutaneously injected into the necks of the mice (6-week-old nude mice). In the control group, normal saline was administered by gavage. In the isoflavone treatment group, isoflavones (160 mg/kg) were administered in the same manner. The dose of isoflavones was based on previous studies [10]. The size of the subcutaneous tumour was measured on days 3, 6, 10, 14, 18, 24, 30, 34, 38, 42, 46, 50, 54 and 58. The mice were humanely euthanized on day 58. Tumor volume was determined by direct measurement with callipers at indicated times and calculated according to the following formula: largest tumour diameter^2^ × second largest tumour diameter × 0.5.

### Statistical analysis

Data were statistically analysed using SPSS Statistics 16.0 software. Results are expressed as mean values ± standard error. Comparisons were made using one-way analysis of variance followed by Dunnett’s test. Significance was determined at a level of *P* < 0.05.
